# An integrated transcriptomic analysis unveils the regulatory roles of RNA binding proteins during human spermatogenesis

**DOI:** 10.3389/fendo.2025.1522394

**Published:** 2025-02-17

**Authors:** Ning Xu, Yixian Qin, Yu Liu, Yudong Guan, Hang Xin, Junwen Ou, Yiqiao Wang

**Affiliations:** ^1^ Centre for Reproductive Medicine, The First Affiliated Hospital of Zhengzhou University, Zhengzhou, China; ^2^ State Key Laboratory of Biotherapy, Sichuan University, Chengdu, China; ^3^ Anti Aging Center, Clifford Hospital, Guangzhou, Guangdong, China; ^4^ Key Laboratory of Regenerative Medicine of Ministry of Education, Institute of Aging and Regenerative Medicine, Department of Developmental & Regenerative Medicine, College of Life Science and Technology, Jinan University, Guangzhou, Guangdong, China

**Keywords:** testis development, spermatogenesis, RNA binding protein, azoospermia, single cell analysis

## Abstract

**Background:**

RNA-binding proteins (RBPs) have emerged as key regulators in testis development and spermatogenesis, yet a comprehensive understanding of their expression dynamics has been lacking.

**Methods:**

This study leverages published single-cell RNA sequencing (scRNA-seq) data to elucidate the complex expression patterns of RBP genes during postnatal testis development and spermatogenesis. Additionally, it uses bulk RNA-seq data to explore the regulatory impact of RBPs on alternative splicing (AS) in non-obstructive azoospermia (NOA).

**Results:**

We have identified cell-specific RNA-binding protein (RBP) genes in various cell types throughout testis development. Notably, distinct RBP gene clusters exhibit significant differential expression, particularly in Sertoli cells as they mature from neonatal to adult stages. Our analysis has revealed temporally-regulated RBP clusters that correlate with the developmental progression of Sertoli cells and the advancement of spermatogenesis. Moreover, we have established links between specific RBPs and the pathogenesis of non-obstructive azoospermia (NOA) through the regulation of alternative splicing (AS) events. Additionally, RPL10, RPL39, and SETX have been identified as potential diagnostic biomarkers for NOA.

**Conclusion:**

This research provided an in-depth look at RBP expression patterns during human testis development and spermatogenesis. It not only deepens our basic comprehension of male fertility and infertility but also indicates promising directions for the creation of innovative diagnostic and treatment methods for NOA.

## Introduction

Postnatal testicular development is a multifaceted and precisely regulated process, predominantly orchestrated by sertoli cells, which govern the differentiation of all the other cell types, including germ cells (1, 2). Following birth, the testicular cord, composed mainly of sertoli and germ cells, gradually evolves into the seminiferous tubule structure, which is encircled by interstitial cells. This structure is paramount for life-long sperm production and androgen secretion. The progression of postnatal testicular development spans across neonatal, infancy, puberty, and adulthood stages, encompassing cellular proliferation, differentiation, and maturation (3).

Spermatogenesis, a complex and highly orchestrated process, unfolds within seminiferous tubules of the testes, with the ultimate goal of generating mature male gametes. This process involves the proliferation of spermatogonia (SPG), differentiation into spermatocytes (SPCs), meiosis to yield spermatids, and the maturation of round spermatids. Sertoli cells, as key drivers of spermatogenesis, provide structural support, establish the blood-testis barrier, and offer immunoprotection, among other essential functions (4–6). Various interstitial cell types complement this process and it is widely acknowledged that these processes are modulated by a spectrum of hormones, mediated by the hypothalamic-pituitary-gonadal axis, as well as other growth factors and cytokines (7–9). Nonetheless, the molecular mechanisms governing postnatal human testis development and spermatogenesis remain elusive.

The mechanism of post-transcriptioinal regulation is well recognized for its crucial role in testis development and spermatogenesis. RBPs constitutet an extensive group of proteins that play predominantly important roles in this regulation through a series of mechanisms, including transcription, alternative splicing, modification, RNA localization, mRNA stability and RNA translation and decay (10). Mouse models have been invaluable in identifying the essential roles of RBPs in nearly all stages of germ-line development from the specification of primordial germ cells (PGC) to the final stage of spermiation (11–14). The complex regulatory networks orchestrated by RBPs are crucial for maintaining cellular homeostasis, and their dysregulation could lead to many pathologies, including non-obstructive azoospermia (NOA) (15). In groundbreaking research, Li Yang and colleagues provided an RBP atlas of mouse male germ cells during spermatogenesis (16). They further discovered that the glutamic acid-arginine patch, a residue-coevolved polyampholytic element present in coiled-coils, can enhance the RNA binding efficiency of its host RBPs. This discovery highlights the nuanced ways in which RBPs can modulate gene expression and suggests that such elements may be important for the precise control of gene expression during spermatogenesis. Due to the marked disparities in reproductive function that exist between humans and rodents, examining RBP expression patterns across human testicular development and spermatogenesis could offer more valuable insights into the molecular mechanisms underlying human spermatogenesis and male fertility.

scRNA-seq has emerged as a powerful tool for dissecting the complexities of biological processes at single cell level (17). This technology has been particularly transformative in the field of testicular biology, significantly enhancing our comprehension of human testicular development and spermatogenesis, as evidenced by several studies (18–25). These investigations have not only mapped the transcriptional profiles across different stages of testicular development but also traced the developmental paths of specific cell types. They have uncovered critical signaling pathways, expanded our knowledge of the expression patterns of key genes, and aided in the identification of markers and genes essential for cell lineage specification, including a number of RNA-binding proteins (RBPs). The rich datasets accumulated through scRNA-seq analyses hold vast potential for re-evaluating cell types, probing into previously unexplored functional aspects, and broadening the range of applications. Scientists have harnessed scRNA-seq to discover new genes, scrutinize their expression patterns, and confirm their roles in cellular processes (26–29). Among these, the research by A.L. Voight is particularly noteworthy for its revelations regarding metabolic changes during human spermatogonial development (30). Despite these significant strides, achieving a thorough molecular understanding of the processes involved in testicular development and spermatogenesis continues to be a fertile ground for further research.

To understand RBP expression during human postnatal testicular development and spermatogenesis, this study utilized available single-cell RNA-seq data, focusing on sertoli cell development and spermatogenesis. By investigating RBP expression profiles, this research aims to unveil specific RBP gene clusters, their dynamic alterations, and potential involvement in NOA. The study strives to deepen our understanding of molecular mechanisms governing human postnatal testicular development and spermatogenesis, particularly concerning NOA pathophysiology.

## Materials and methods

### Retrieval and processing of scRNA-seq data

We obtained the Unique Molecular Identifier (UMI) count matrix from the Gene Expression Omnibus (GEO) datasets GSE124263, GSE149512, GSE134144, and GSE112013, encompassing single-cell RNA-seq data from 17 human testis samples across four distinct age groups (**Table 1**). This UMI count matrix underwent transformation into a Seurat object using the R package Seurat (31) (version 4.0.4). Cells with UMI counts <1000, genes detected in fewer than 500 cells, or displaying over 25% mitochondrial-derived UMI counts were flagged as low-quality cells and subsequently removed. Genes detected in fewer than five cells were excluded from subsequent analyses.

**Table 1:** The clinicopathological features of the cohorts enrolled in this study.

	Samples for immunohistochemistry						GSE190752						GSE124263		GSE149512			GSE149512	GSE134144		GSE149512						GSE112013		
Individual	NOA-1	NOA-2	NOA-3	OA	OA	OA	NOA-1	NOA-2	NOA-3	control-1	control-2	control-3	Neonate		Infancy			Puberty			Adult								

Age	27 years	26 years	32 years	26 years	35 years	27 years	38 years	28 years	33 years	24 years	26 years	29 years	2 days	7 days	2 years	5 years	8 years	11 years	11 years	13 years	17 years	23 years	25 years	28 years	28 years	31 years	17 years	24 years	25 years
BMI	27.8	21.9	23.6	23.8	26.8	31.3	25.22	22.31	27.09	28.85	26.32	21.62																	
Testicular volume (Left/Right, ml)	10/10	6/6	8/8	15/15	12/12	12/15	10/10	12/12	10/10	15/15	12/12	12/12																	
Somatic karyotype	46,XY	46,XY	46,XY	46,XY	46,XY	46,XY	46,XY	46,XY	46,XY	46,XY	46,XY	46,XY																	
Y Chromosome microdeletions	No	No	No	No	No	No	No	No	No	No	No	No																	
Sex hormone																													
Follicle-stimulating hormone (mIU/ml)	14.12	23.67	10.67	3.26	6.67	2.27	37.21	16.22	8.15	1.58	2.28	7.41																	
Luteinizing hormone (mIU/ml)	8.19	8.66	7.39	3.35	2.09	3.5	9.2	4.42	2.57	9.68	1.13	5.27																	
Testosterone (ng/ml)	7.41	3.38	1.52	3.73	2.86	3.28	7.04	2.91	3.64	3.59	7.51	3.77																	
Estradiol (pg/ml)	43.02	94.5	28.67	20.46	34.3	32.52	48.5	27.25	12	N/A	32.15	40.33																	
Prolactin (ng/ml)	23.47	13.76	4.57	10.76	4.48	15.7	9.36	14.33	15.1	N/A	20.03	12.26																	
History of previous pregnancies	No	No	No	No	No	No	Induced abortion	Having a child	Induced abortion	No	No	No																	
Jonhsen Score	6	6	6	N/A	N/A	N/A	N/A	N/A	N/A	N/A	N/A	N/A																	

GSE124263: These testes were from unrelated day 2 and day 7 neonates who died as a result of nontesticular-related medical dysfunction.

GSE149512: Ten normal samples (5 underage and 5 OA donors) all had normal karyotypes, genotypes, sex hormone levels, and morphology of seminiferous tubules according to their age. 5 donors samples (2-17 years) were obtained when they underwent testicular biopsy or partial excision and 5 OA samples(23-31 years) were obtained from the abandoned tissues after testicular sperm extraction operation.

GSE134144: Those human testicular samples were removed from deceased individuals who consented to organ donation for transplantation and research.

GSE112013: Those samples were removed from deceased individuals who consented to organ donation for transplantation and research.

### scRNA-seq data preprocessing and quality control

Following quality control, log normalization was applied to the UMI count matrix. To establish potential Anchors for data integration, the top 2000 variable genes were identified using the FindIntegrationAnchors function in Seurat. Subsequently, the IntegrateData function was employed to integrate the datasets. Principal Component Analysis (PCA) was conducted on the integrated data matrix to reduce dimensionality. The Elbowplot function in Seurat determined the top 50 principal components for downstream analyses. Major cell clusters were identified using the FindClusters function in Seurat (resolution set to default - res = 0.6). These cells were then grouped into 25 distinct cell types and visualized using Uniform Manifold Approximation and Projection (UMAP) plots. To assign cell types to each cluster, gene markers were identified using the “FindAllMarkers” function in Seurat (v4.0.4) with specified parameters. Further cell type annotation utilized ScType tools (32), employing previously published testis marker genes (18, 33, 34).

### Analysis of RBP genes

A catalog comprising 2,141 RBPs retrieved from four previous reports (35–38) was utilized. The UMI count matrix of RBPs served as input for Seurat to determine cell clusters, and differentially activated RBPs were selected using the “FindAllMarkers” function.

### scRNA-seq differential gene expression analysis

Differentially expressed genes (DEGs) were identified using the FindMarkers/FindAllMarkers function from the Seurat package (one-tailed Wilcoxon rank sum test, p-values adjusted using the Bonferroni correction). Genes exhibiting a natural log scale expression difference of at least 1 and adjusted p-value < 0.05 were considered as DEGs.

### Time course seq analysis

TCseq (https://bioconductor.org/packages/release/bioc/html/TCseq.html) was employed to assess trends in RBP expression across different ages of sertoli cells, evaluating the average expression level of differentially expressed RBPs between any two age groups. RBPs were clustered into 8 groups based on similar expression patterns.

### Pseudotime trajectory analysis using monocle3

Monocle3 (39) (v1.0.0) was employed to unveil the pseudotime trajectory in germ cells. Dimensionality reduction and trajectory analysis were conducted using standard workflows and default parameters.

### Retrieval and processing of bulk RNA-seq data

Public sequence data files from GSE190752 were downloaded from the Sequence Read Archive (SRA). SRA Run files were converted to fastq format using NCBI SRA Tool fastq-dump (v.2.8.0). The raw reads were subjected to quality trimming using FASTX-Toolkit (v.0.0.13; http://hannonlab.cshl.edu/fastx_toolkit/). Clean reads were evaluated using FastQC (http://www.bioinformatics.babraham.ac.uk/projects/fastqc).

### Bulk-RNA-seq reads alignment and DEG analysis

Clean reads were aligned to the human genome with HISAT2 (40). Uniquely mapped reads were used to calculate read number and Fragments Per Kilobase of exon per Million fragments mapped (FPKM) for each gene. DEseq2 (41) was applied to identify DEGs based on fold change (FC≥2 or ≤0.5) and false discovery rate (P value ≤ 0.05). The expression profile of differentially expressed RBPs was filtered from all DEGs using a catalog of 2,141 RBP genes from previous reports (35–38).

### Alternative splicing analysis

Regulatory Alternative Splicing events (RAS) were defined and quantified using the Splice site Usage Variation Analysis (SUVA) pipeline (42). Analysis of different splicing for each group was conducted, calculating Reads Proportion of SUVA AS event (pSAR) for each AS event.

### Co-expression analysis

Co-expression analysis was performed for all differentially expressed RBPs and RAS (pSAR≥50%). Pearson correlation coefficients between differentially expressed RBPs and RAS were calculated, screening differentially expressed RBP-RAS relationship pairs satisfying an absolute correlation coefficient ≥0.99 and P value ≤ 0.01.

### Functional enrichment analysis

Gene Ontology (GO) terms and KEGG pathways were identified using KOBAS 2.0 (43) to categorize functional gene categories. Hypergeometric tests and Benjamini-Hochberg FDR controlling were utilized for term enrichment.

### Other statistical analysis

The heatmap package in R was employed for clustering based on Euclidean distance.

### Clinical sample collection

This study was conducted according to the guidelines of the Declaration of Helsinki and approved by the Ethics Committee of Scientific Research and Clinical Trial of the First Affiliated Hospital of Zhengzhou University(protocol code YFSZ-2024-020). The paraffin-embedded testicular biopsies of three obstructive azoospermia (OA) and three NOA patients were provided by the department of pathology, the first affiliated hospital of zhengzhou university. The age of the donors ranged from 22 to 30 years old. The NOA patients all have a Johnsen’s Score 6 (**Table 1**).

### Immunohistochemical staining

The testicular slides were de-paraffinized in xylene and then added to the ethanol following the below concentrations: 100% ethanol (3 min), 100% ethanol (3 min), 85% ethanol (3 min), 75% ethanol (3 min). Subsequently, the slides were repaired in boiling EDTA solution(Servicebio,G1203-250ML) for 10 minutes. After washing the slides three times in phosphate buffered saline (PBS), 5 minutes each, we incubated these slides in 3% hydrogen peroxide in endogenous peroxidase blocking buffer in room temperature for 20 minutes. Non-specific binding were blocked in PBS supplemented with 3% bovine serum albumin (BSA) for 30 minutes at 37°C. Slides were incubated with the primary antibody at 4°C overnight and then incubated with the secondary antibody for 30 minutes at 37°C. Peroxidase activity was detected by 3,30 -diaminobenzidine tetrahydrochloride (DAB) kid (Zli-9018) and nuclei were counterstained with hematoxylin. After dehydration, a coverslip was placed on the slides with mounting medium. Images were obtained by a microscope (Nikon, 90i, Tokyo, Japan). The antibodies used were as follows: Rabbit anti-RPL10 (1:200, Proteintech, 17013-1-AP), Rabbit anti-RPL39 (1:100, Proteintech, 14990-1-AP), Rabbit anti-SETX (1:100, CSB-PA800097LA01HU), Goat-anti-mouse/rabbit IgG(ZSGB-BIO, PV-6000).

## Results

### Profiling cell diversity across human postnatal testis development

**Figure 1:**
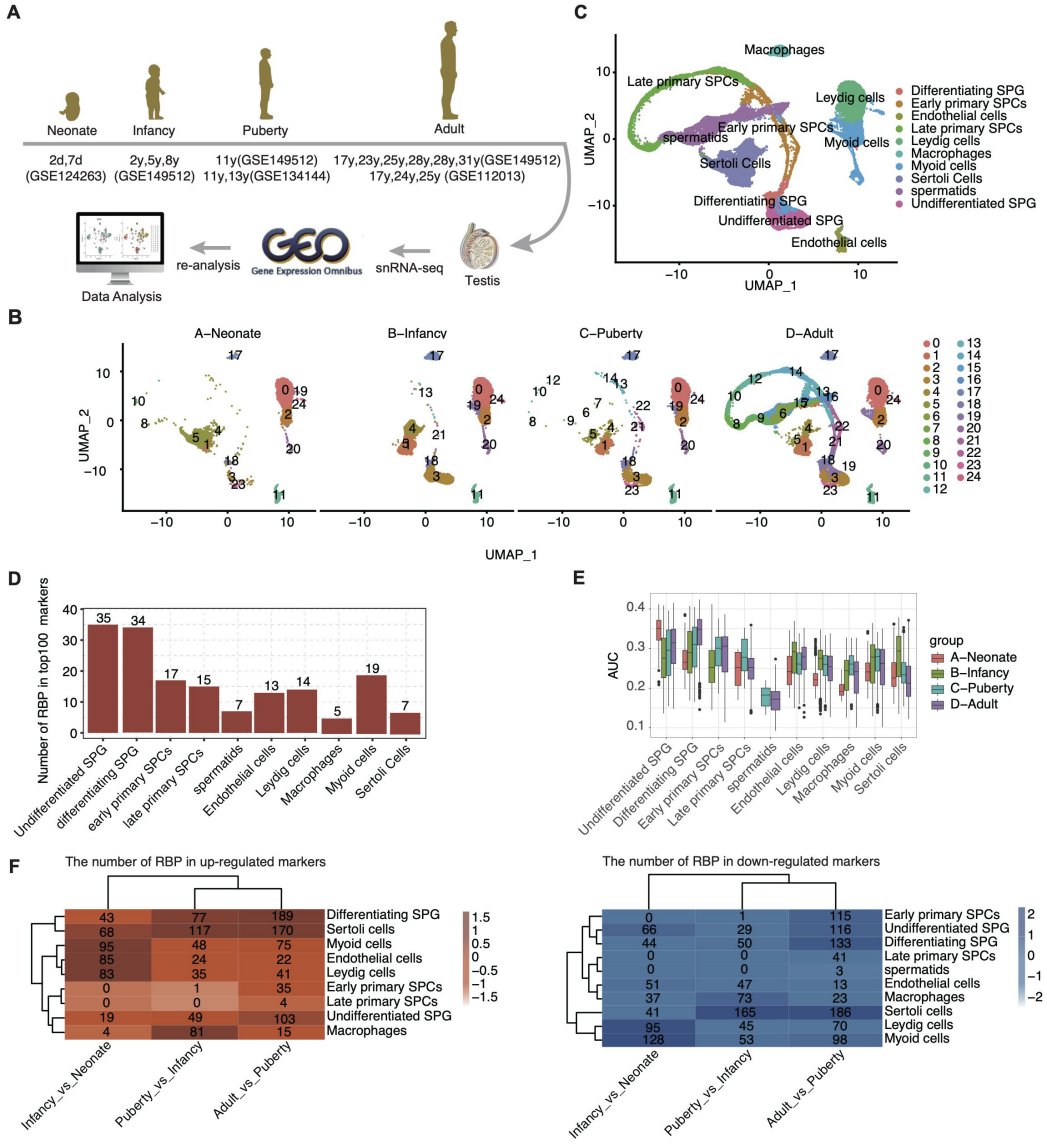
ScRNA-seq analysis of human testis from different development stages identified distinct cell types. **(A)** Schematic illustration of scRNA-seq data selection and processing. **(B)** UMAP plot of composite single-cell transcriptomic profiles from all samples from different age groups. **(C)** UMAP plot of composite single-cell transcriptomic profiles. The 10 cluster identities were assigned based on the expression patterns of known marker genes. **(D)** Bar plot showing the number of RBP in top100 marker genes in each cell type. **(E)** Box plot comparing the AUC score of each cell type within each sample group. **(F)** The heatmap displays the number of RBPs for each cell type across different age groups.

To delineate the cellular landscape during human postnatal testicular development, we systematically curated single-cell transcriptome datasets from four distinct stages, as represented by NCBI GEO entries: neonate (postnatal day 2 and 7), infant (2, 5, 8 years), puberty (11-13 years), and adulthood (17-31 years). After rigorous quality control, a total of 67,400 high-quality single cells were retained for further analyses (**Figure 1A**).

Employing unbiased clustering analysis and UMAP for dimensionality reduction, the integrated datasets yielded 25 distinct cell clusters (**Figure 1B**). Utilizing the SCTYPE software in conjunction with established cell-specific markers (**Supplementary Figure S1A**), we identified 10 predominant testicular cell types (**Figure 1C**). These included various stages of germ cells, such as undifferentiated SPG, differentiating SPG, early and late primary SPCs, and spermatids. Additionally, prominent somatic cell types were delineated, including sertoli cells, leydig cells, macrophages, myoid cells, and endothelial cells.

Further analysis of the distribution of cell types across developmental stages revealed a significant increase in the proportion of undifferentiated SPG upon entering the infant stage (**Supplementary Figure S1C**). Each cell type expressed specific marker genes, with the top three listed for each (**Supplementary Figure S1A**). The functional pathways enriched by these marker genes were consistent with the known functions of the respective testicular cell types (**Supplementary Figure S1B**).

Among the cell-type specific genes, a substantial proportion was identified as RBPs, particularly within undifferentiated SPG (35 out of 100) and differentiating SPG (34 out of 100) (**Figure 1D**). These two cell types also displayed higher Area Under the Curve scores for RBP expression (**Figure 1E**). Interestingly, the expression pattern of RBPs across different cell types exhibited dynamic changes with development (**Figure 1F**, **Table 2**).

**Table 2:** DEGs in cell types, |ave_logFC| > 0.5, adj_p value < 0.05.

Cluster	B-Infancy_vs_A-Neonatal (up)	B-Infancy_vs_A-Neonatal (down)	C-Puberty_vs_B-Infancy (up)	C-Puberty_vs_B-Infancy (down)	D-Adult_vs_C-Puberty (up)	D-Adult_vs_C-Puberty (down)
Differentiating SPG	186	204	225	153	725	321
Early primary SPCs	0	0	3	22	214	262
Endothelial cells	375	206	152	190	141	117
Late primary SPCs	0	0	0	0	95	197
Leydig cells	410	232	199	139	264	204
Macrophages	44	81	276	336	108	98
Myoid cells	455	278	232	179	394	315
Sertoli Cells	252	183	382	624	954	461
spermatids	0	0	0	0	0	45
Undifferentiated SPG	77	285	125	115	465	246

### Profiling cell type-specific RBPs in human testis

**Figure 2:**
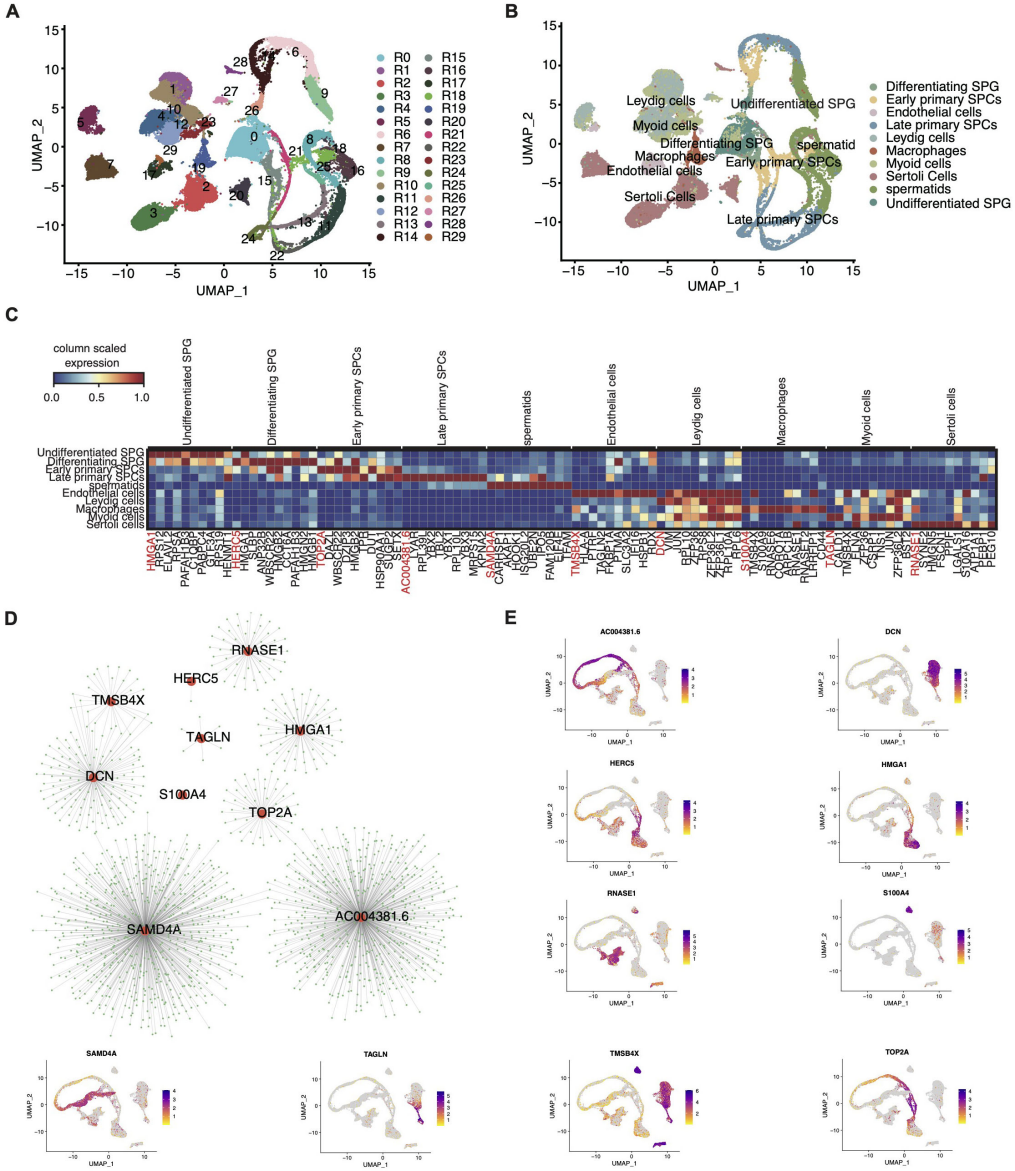
Comprehensive single-cell transcriptome analysis reveals a large number of RBPs specifically expressed in different cell types of testis. **(A)** UMAP plot of scRNA-seq profile based on RBP expression module. **(B)** UMAP plot of scRNA-seq profile based on the known marker genes. **(C)** UMAP showing relative expression (z score, column scaled) levels of top10 RBP markers of each cell type in single-cell dataset. **(D)** Cytoscape shows the co-expression networks comprising 10 RBPs selected from 10 cell types and target genes. **(E)** Gene expression level of 10 RBPs was represented with UMAP plot.

Given the established importance of RBPs in spermatogenesis (11–13), we pursued a focused investigation into the role of RBPs within the testicular cell atlas. Based on a curated list of 2141 RBPs genes from prior studies, we performed re-clustering of these cells. Unsupervised clustering with UMAP visualization revealed 30 clusters with similar RBPs expression patterns (**Figure 2A**). These RBP clusters showed a high degree of specificity for distinct cell types (**Figure 2B**), with distinct RBP marker genes associated with each cluster presented in **Supplementary Figure S2A**.

Our analysis indicated that most cell types predominantly exhibited one to two primary RBP clusters. For example, undifferentiated SPG prominently expressed R0, macrophages exhibited R19, and endothelial cells displayed R16 as the primary RBP clusters (**Supplementary Figure S2E**). Notably, with increasing age, certain cell types displayed significant alteration in the composition of RBP clusters. For instance, sertoli cells transitioned from cluster R8 in neonates to R1, R3, and R6 during infancy and puberty, ultimately stabilizing at R19 and R26 in adulthood (**Supplementary Figure S2E**). Additionally, the overall heterogeneity of RBP clusters in the testis increased with age (**Supplementary Figure S2B**).

Moreover, we identified cell-specific RBP genes across various developmental stages and cell types, which may play crucial roles in their respective cell biology. Consistent with prior reports, numerous cell-specific RBPs were identified in germ-line cells, including undifferentiated SPG, differentiating SPG, early and late SPCs, and spermatids (**Supplementary Figure S2C**). The top ten cell-specific RBP genes for each cell type are outlined in **Figure 2C**. Interestingly, some of these genes, like *ELAVL2* specifically expressed in SPG, corroborate previous findings (18).

We further constructed co-expression networks of the top specific RBP genes in each cell type (**Figures 2D, E**) and performed Gene Ontology (GO) analysis (**Supplementary Figure S2D**). Collectively, our study presents a comprehensive profile of cell type-specific RBPs in the human testis, shedding light on their potential regulatory roles in testicular cell biology.

### Heterogeneity and regulatory modules of development-related RBPs revealed in sertoli cells

**Figure 3:**
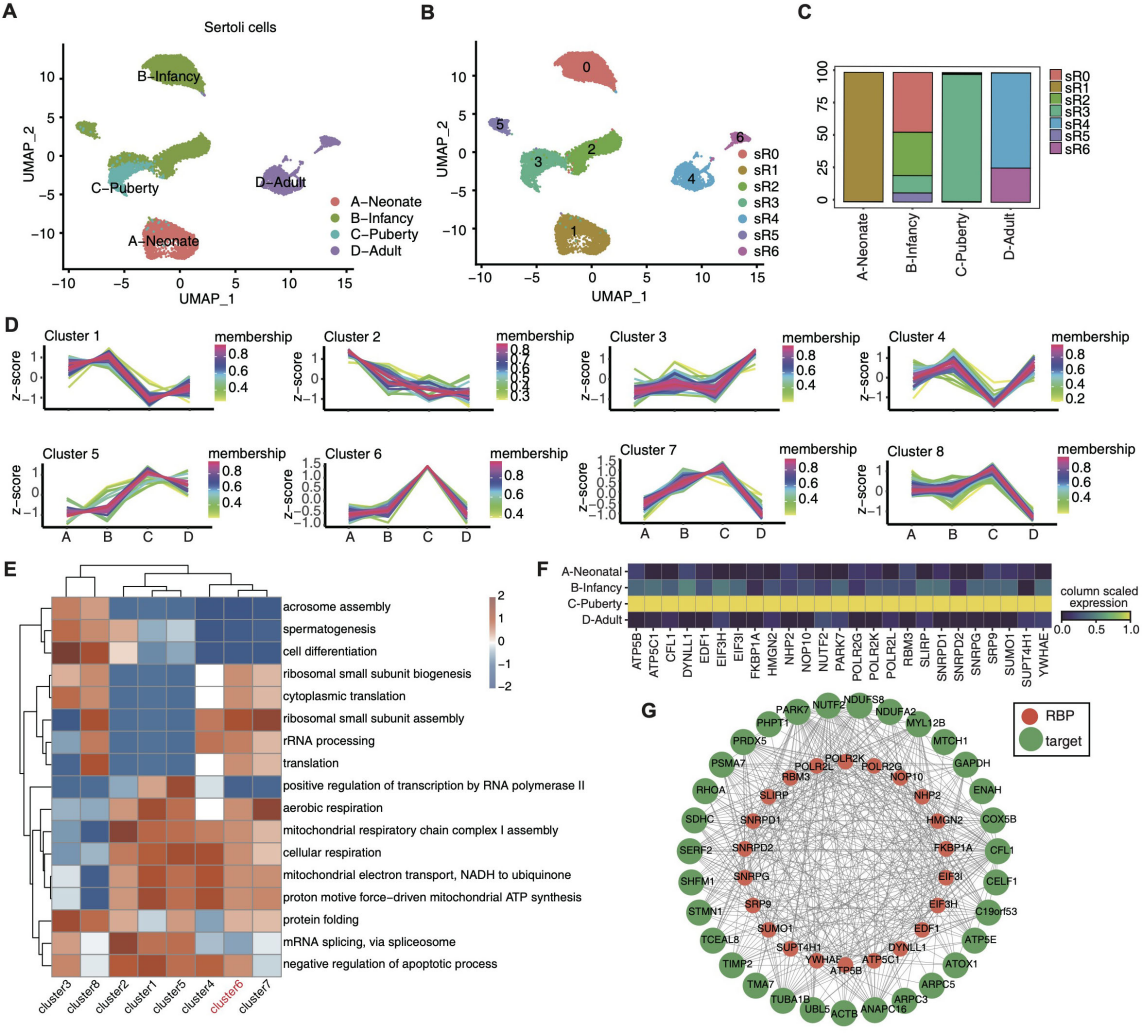
Single-cell analysis revealed heterogeneity and regulatory module of development-related RBPs in sertoli cells. **(A, B)** UMAP was reperformed to display the distributions of 7 cell clusters based on RBP expression module using sertoli cells. **(C)** Stacked bar plot showing the relative proportions of RBPs expression module in different age groups. **(D)** Dynamic cluster analysis of the RBP expression in sertoli cells across various age groups based on TCseq. **(E)** Gene ontology enrichment analysis of biological processes of target genes associated with RBPs belonging to different clusters by TCseq. Top 5 terms were selected for each cluster and heatmap shows the enrichment q-value of these terms (scaled by column). **(F)** Unsupervised clustering heatmap showing relative expression (z score, column scaled) levels of RBP genes in Cluster 6 showed in E, according to different age groups. **(G)** Cytoscape shows the co-expression networks comprising RBP genes from cluster 6 and their target genes.

Considering the pivotal role of sertoli cells in testis development, we focused on the dynamic changes in RBPs across different developmental stages within sertoli cells. Focusing on sertoli cell datasets, we conducted unsupervised clustering analysis. The UMAP uncovered 7 distinct cell clusters (**Figures 3A, B**), each characterized by specific RBP marker genes (**Supplementary Figure S3A**). The distribution of these RBP clusters across various developmental stages is depicted in **Figure 3A**, highlighting pronounced stage-specific characteristics.

Each developmental stage prominently displayed one to two dominant RBP clusters, such as R1 in neonate, R0, R2, R3and R5 in infancy, R3 in puberty, and adulthood with R4 and R6 (**Figure 3C**). We investigated the dynamic alterations of RBPs within sertoli cells across different developmental stages by identifying differentially expressed RBPs between adjacent time points and subjecting them to TC seq analysis, which unraved 8 time-dependent RBP clusters (**Figure 3D**). The expression pattern of gene cluster 6 shown a sharp increase in purbery and remained low in other three stages, suggesting its potential role in driving sertoli cell maturation. The biological functions of these gene clusters were evaluated via GO enrichment analysis (**Figure 3E**), with gene cluster 6 notably enriched in ribosomal small subunit biogenesis, cytoplasmic translation, rRNA processing, translation and aerobic respiration, etc. Cluster 6 pathway-related genes were shown in **Figure 3F**, including *ATP5B,CFL1,DYNLL1,EDF1,EIF31*, etc.

Furthermore, we performed co-expression analysis between these RBP genes and their target genes (**Figure 3G**), with UMAP visualization demonstrating the expression profiles of RBM3, NOP10, PAPK7 and FKBP1A across distinct developmental stages (**Supplementary Figure S3B**). The function of these target genes are involved in cell proliferation and differentiation(e.g., CELF1 and PSMA7), cytoskeletal reorganization(e.g.,ARPC3 and CFL1), energy metabolism (e.g.,NDUFS8 and SDHC), and cell cycle regulation (e.g., STMN1 and TUBA1B).

### Profiling development-associated RBP modules in spermatogenesis

**Figure 4:**
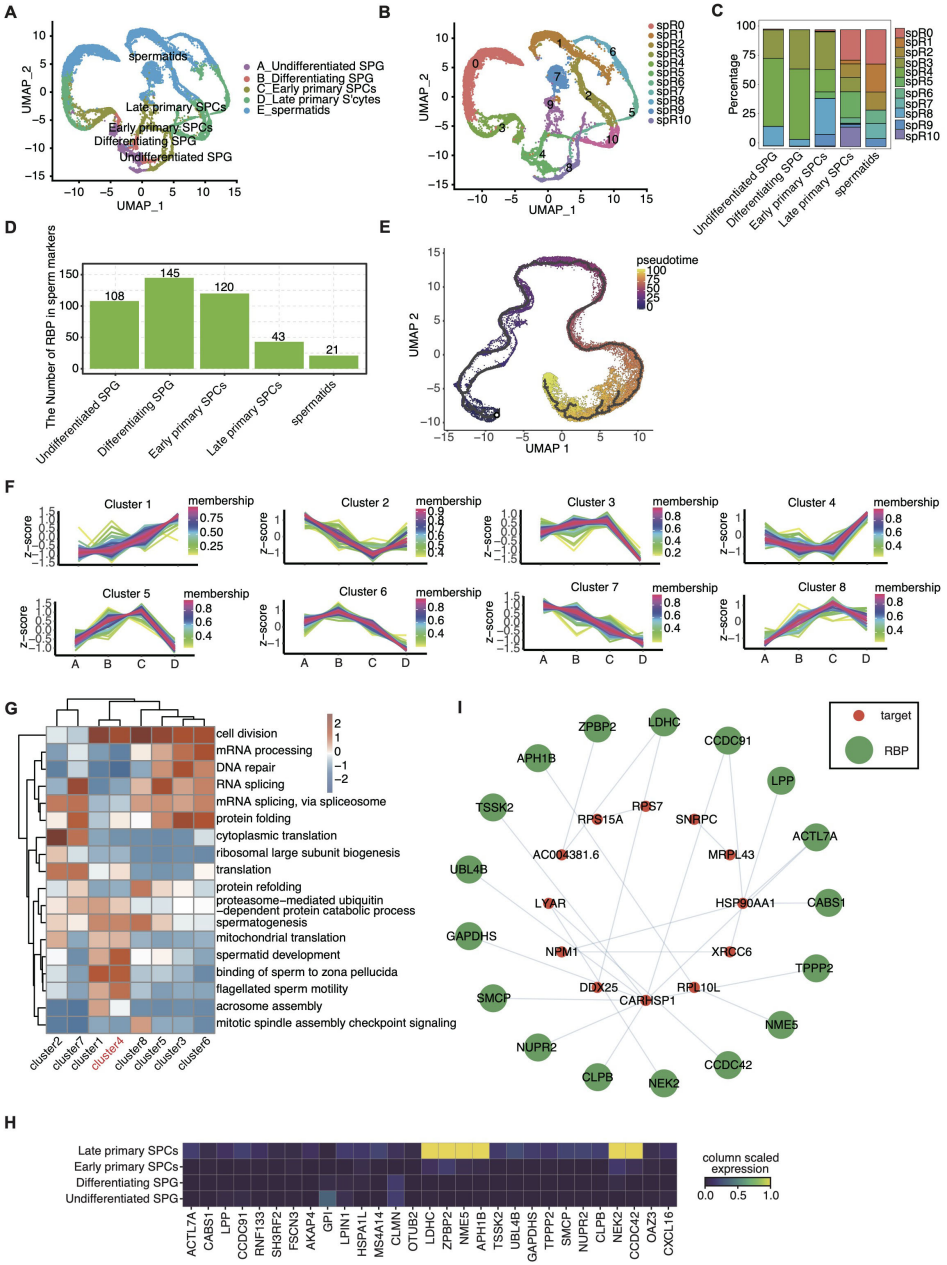
Identification of RBP modules in spermatogenesis. **(A)** UMAP displays the distribution of 5 cell types based on germ cells. **(B)** UMAP was reperformed to display the distributions of 11 cell clusters based on RBP expression module. **(C)** Stacked bar plot showing the relative proportions of RBPs expression module in different age groups. **(D)** Bar plot showing the number of RBP in marker genes of each cell type. **(E)** Pseudotime analysis of all germ cells. **(F)** Dynamic cluster analysis of the RBP expression in germ cells across the four development stages based on TCseq. **(G)** Gene ontology enrichment analysis of biological processes of genes involving target genes of RBPs belonging to different clusters by TCseq. Top 5 terms were selected for each cluster and heatmap shows the enrichment q-value of these terms (scaled by column). **(H)** UMAP showing relative expression (z score, column scaled) levels of RBP genes involved in cluster 4 showed in **(G)**, according to different germ cell types. **(I)** Cytoscape shows the co-expression networks comprising target genes selected from cluster 4 associated with spermatogenesis and RBPs.

Recognizing the pivotal role of RBPs in spermatogenesis and their implications in male infertility, we aimed to delineate the landscape of RBP expression in germ cells throughout spermatogenesis. Focusing on adult-stage germ cell datasets, unbiased clustering analysis revealed a clear UMAP embedding representation that captured the dynamic progression of germ cells from undifferentiated SPG to spermatids (**Figure 4A**).

Additionally, leveraging the RBP genes, we reclustered these germ cells, identifying 11 distinct RBP-based cell clusters (**Figure 4B**). Each distinct RBP cluster exhibited specific RBP marker genes, with the top 3 markers highlighted in **Supplementary Figure S4B**. Intriguingly, these RBP clusters exhibited dynamic changes throughout the differentiation process. Specifically, the RBP clustering composition displayed a notable transition (**Figure 4C**), indicating a close association between undifferentiated SPG, differentiating SPG, and early SPCs, while late SPCs and spermatids showed a higher similarity. Importantly, our analysis revealed a higher prevalence of cell-specific RBPs during the early stages of spermatogenesis, as depicted in **Figure 4D**. The top 10 RBP mark genes in each cell types were shown in **Supplementary Figure S4A**.

Standard trajectory analysis only reveals genes associated with differentiation, but does not establish causality. RBPs have been demonstrated as key upstream regulators during stem cell differentiation (44, 45), thus we perform Pseudotime Trajectory Analysis by Monocle3 based on RBPs and depicted the sequential changes in germ cells during spermatogenesis (**Figure 4E**). Subsequently, we investigated the dynamic alterations in RBP gene expression during spermatogenesis, identifying eight clusters with distinct time-dependent expression patterns (**Figure 4F**). The biological functions of genes within these clusters were also assessed, demonstrating associations with key terms such as spermatogenesis, spermatid development, RNA splicing, mRNA splicing, mRNA processing and cytoplasmic translation, etc. (**Figure 4G**).

As shown in **Figure 4F**, the expression pattern of gene cluster 4 shown significant increase in late primary SPCs, while gene cluster 3 exhibited the opposite trend, indicating their potential role in late meiosis. Their expression profiles across various cell types were delineated, constructing co-expression networks with target genes within germ cells (**Figures 4H, I**, **Supplementary Figures S4C, D**). These results provide a comprehensive overview of RBP profiles and reveal the intricate regulatory patterns of RBPs throughout spermatogenesis, laying the foundation for further molecular mechanism exploration.

### Dysregulated cell-type specific RBPs and their implication in splicing aberrations associated with NOA

**Figure 5:**
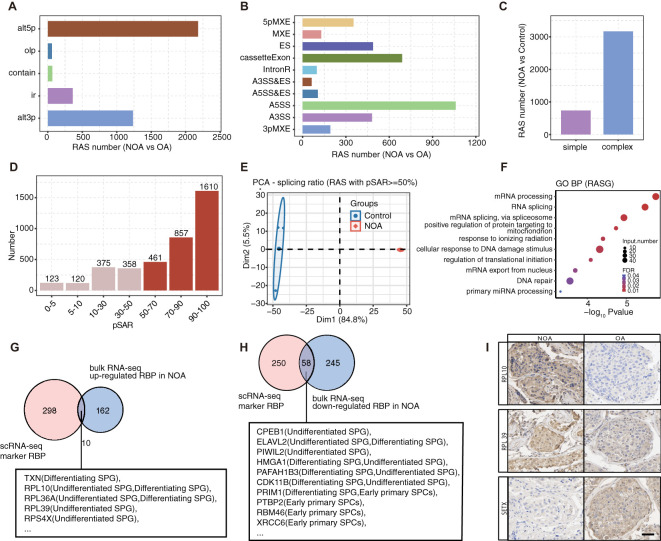
Cell type-specific RBPs associated with spermatogenesis and related RAS events are significantly co-disturbed in testis tissues of patients with NOA and OA. **(A)** Five different types of AS event model defined by SUVA according to splicing site usage variation. Each type contains two paired splice junctions (SJ) (for “ir” type paired SJs is one SJ). “alt3p” indicates model that 5’ splice site is shared and 3’ splice site is alternative. “alt5p” indicates model that 3’ splice site is shared and 5’ splice site is alternative. “olp” indicates model that both splice sites are different but part of the splice junction are overlapped. “contain” indicates model that both splice sites are different but one splice junction is contained in another splice junction. “ir” indicates model that a pair of splice sites are either used or not used which is identical to the “intron retention” event in classical definition. Bar plot showing number of regulated RAS detected by SUVA between NOA and OA samples. **(B)** Splice junction constituting RAS event detected by SUVA was annotated to classical AS event types. And the number of each classical AS event types were showed with bar plot. **(C)** Bar plot showing number of SUVA RAS events contains SJs involved in two or more different classical splicing events (complex) of in same classical splicing event (simple). **(D)** Bar plot showing RAS number with different abundance (pSAR) of all detected RAS. RAS with pSAR>=50% were used for further analysis and were filled with dark red color. **(E)** PCA of splicing ratio of RAS which pSAR >= 50%. The ellipse for each group is the confidence ellipse. **(F)** Top10 enriched GO biological process terms of genes involved RAS with pSAR >= 50%. **(G, H)** Venn diagram showing the overlap of DE RBPs from bulk RNA-seq and cell type specific RBPs of germ cells identified in scRNA-seq dataset. **(I)** In comparison with the OA, there were significant up-regulation in the protein expression levels of RPL10 and RPL39 in testicular samples from NOA patients. Conversely, the protein expression levels of SETX were notably down-regulated in the same testicular samples from NOA patients.

The significance of AS in spermatogenesis has been recognized for several decades (46). RBPs are key regulators of AS and we hypothesize that their dysregulation may precipitate AS irregularities, consequently leading to impaired spermatogenesis, like NOA. In this study, we conducted SUVA on bulk-seq data from three patients with NOA and three patients with obstructive azoospermia (OA) (26). Bulk RNA-seq generates an averaged transcriptome across all constituent cells. However, it offers profound sequence depth, facilitating the comprehensive capture of the maximal number of genes and alternative splice variants. Our results revealed thousands of significantly differential splicing events, encompassing various types such as alternative 5’ splice site (alt5p), alternative 3’ splice site (alt3p), intron retention (ir), and other less frequent splicing types (**Figure 5A**). Notably, more than 75% of NOA-associated splicing alterations were complex splicing events, highlighting the intricate nature of AS regulation in NOA (**Figures 5B, C**).

Focusing on high-frequency and dominant RBP-associated splicing (RAS) events (**Figure 5D**), we observed clear distinctions between the NOA and OA groups in principal component analysis based on splicing ratios (**Figure 5E**). Further investigation into 2928 dominant RAS-related genes revealed enrichments in essential pathways like mRNA processing, RNA splicing, and DNA damage response pathways (**Figure 5F**, **Supplementary Figure S5C**).

Subsequently, we identified differentially expressed RBPs between NOA and OA patients, predominantly observing downregulation, suggesting the potential inactivation of RBPs in NOA pathogenesis (**Supplementary Figure S5D**). By overlapping cell-type specific RBPs from scRNA-seq data with differentially expressed RBPs from bulk RNA data, we pinpointed 10 upregulated and 58 downregulated RBPs, primarily expressed in early spermatogenesis stages (e.g., undifferentiated SPG, undifferentiated SPG, and early SPCs among the overlapping sets) (**Figures 5G, H**). Consequently, we investigated the differential protein expression of *RPL10*, *RPL39* and *SETX* between OA and NOA patients using immunohistochemistry staining (**Figure 5I**). We found that RPL10 and RPL39 are primarily localized in the cytoplasm, while SETX is localized in both the nucleus and the cytoplasm. In the testicular tissue of OA, RPL10, RPL39, and SETX are expressed in spermatogonia, early and late spermatocytes, as well as Sertoli cell. Additionally, we observed differential expression in NOA, which is consistent with the mRNA data from single-cell sequencing.

We further conducted Pearson’s correlation analysis between these differentially expressed RBPs and spermatogenesis and spermatid development pathway-related NOA-RAS, revealing a strong correlation (correlation >= 0.99, p value <= 0.01). This analysis allowed the prediction of the potential regulatory role of these RBPs in AS. A coherent co-disturbed network was constructed to visualize the relationship between the overlapped RBPs and spermatogenesis pathways-related NOA-RAS, suggesting a potential regulatory influence of these RBPs on NOA-associated splicing alterations (**Supplementary Figure S5F**).

In summary, our findings elucidate dysregulated cell-type specific RBPs in NOA and predict their potential role in AS regulation, providing novel insights into the molecular mechanisms underlying NOA and offering a prospective avenue for utilizing these RBPs as biomarkers for NOA.

## Discussion

This study represents a significant advancement in the field by integrating scRNA-seq datasets across the human lifespan, with a particular emphasis on RBPs. Our findings contribute three novel insights to the existing literature: 1) The identification of cell-type specific and stage-specific RBP gene clusters, revealing differential expression patterns across various cell types during human postnatal development and spermatogenesis; 2) The observation of dynamic changes in RBP expression within sertoli and germ cells as they transition through distinct developmental stages; and 3) The revelation of RBPs’ role in the pathogenesis of NOA through the regulation of AS.

Our research underscores the complexity and dynamism of testis development and spermatogenesis in humans, processes that have been extensively studied in rodents but remain inadequately characterized in humans. The recent adoption of scRNA-seq technology has begun to bridge this knowledge gap, providing a more nuanced understanding of human testis development, spermatogenesis, and conditions such as NOA (47, 48). Despite the generation of comprehensive cellular atlases, the molecular mechanisms and regulatory pathways, particularly the post-transcriptional processes mediated by RBPs, remain elusive. Our study leverages the extensive datasets generated by scRNA-seq, coupled with sophisticated bioinformatic analyses, to uncover specific RBPs that are likely to regulate spermatogenesis at different life stages.

Our study’s contribution lies in the re-analyzing and integration of published human testis scRNA-seq data, presenting for the first time a detailed landscape of RBP expression patterns during postnatal development and spermatogenesis. By meticulously profiling 67,400 single cells post quality control and classifying 10 major cell types based on distinct marker genes, we have provided a robust foundation for further investigation. GO analysis further confirmed the enrichment of functional pathways associated with specific cell functions, highlighting the significance of RBP genes in cell type-specific functions, notably in undifferentiated SPG and differentiating SPG. This aligns with previous rodent studies (49) and underscores their pivotal roles in spermatogonial proliferation and differentiation.

Our examination of global RBP expressions across various cell types during testis development unveiled 29 distinct RBP clusters with cell type-specific genes, demonstrating the complex regulatory dynamics during development. The increasing heterogeneity of RBP clusters in the testis highlights the intricate regulatory mechanisms at play. For instance, Sertoli cells, which play a pivotal role in supporting germline development (6), exhibited RBP expression patterns that are specific to certain stages of development, indicating their critical involvement in the maturation process of the testis. In contrast, macrophages and endothelial cells showed stable RBP patterns, suggesting a more stable and less developmentally dynamic role.

In the postnatal development of the testis, Sertoli cells are pivotal in nurturing germ cell development (6). Despite the active transcriptional activities within Sertoli cells, our comprehension of post-transcriptional regulation in these cells remains limited. Previous research has charted the developmental trajectory of Sertoli cells, revealing that two types of immature Sertoli cells evolve into a single mature form (33). Our study’s identification of six distinct clusters of Sertoli cells across various developmental stages, based on the expression of RNA-binding proteins (RBPs), not only points to different functional states but also suggests that Sertoli cell maturation is a gradual process. This finding underscores the importance of RBP expression patterns across different postnatal stages, particularly the notable increase in cluster 6 RBP genes during puberty, which may significantly influence Sertoli cell maturation. The target genes of these RBPs are involved in cell proliferation and differentiation, cytoskeletal reorganization, energy metabolism and cell-cycle regulation, all of which are potentially crucial for Sertoli cell maturation.

Our identification of numerous specific RBP genes in each germ cell type, some previously implicated in spermatogenesis (50, 51), strengthens their significance and provides a basis for future functional studies. Our findings underscore the developmental shifts in RBP expression and reveal distinct dynamic patterns of RBP modules during spermatogenesis, aligning with previous studies on rodents (16). In addition, we have primarily investigated the target genes of these RBPs, some of which are associated with spermatogenesis, while further research into RBP interaction networks and underlying molecular mechanisms could provide more comprehensive insights. Nonetheless, our study represents the initial effort to systematically understand the activities of RBPs in human spermatogenesis.

In exploring the application of RBPs in NOA, our study highlighted the association between AS events and dysregulated RBPs. NOA, characterized by a severe reduction or absence of multiple types of germ cells, remains poorly understood in terms of its etiology and underlying pathological mechanisms. Our identification of 63 differentially expressed RBPs in NOA, including known spermatogenesis-related genes like RBM46, SAMD4a, HMGA1, PIWIL1, DDX25, and HENMT1 (52–55), provides new avenues for understanding the molecular mechanisms underlying this condition. The mutation of DDX25, for example, has been associated with spermatogenic failure and NOA in humans (55). Our validation of the differential expression of proteins RPL10, RPL39, and SETX in clinical samples aligns with findings from rodent studies (56–58), suggesting their potential as diagnostic biomarkers for NOA.

The complexity of AS events in NOA cases, with over 75% being complex AS events, indicates the intricate regulation of spermatogenesis-related genes. Our study unveils the potential regulatory roles of RBPs in AS within the context of NOA, offering insights into the molecular mechanisms underlying this condition and highlighting the need for further investigation into the targets and associated regulatory pathways of RBPs.

In conclusion, our work presents a comprehensive landscape of RBP expression in postnatal testis development, with a focus on sertoli and spermatogenic cells, and implicates RBPs in NOA pathogenesis through AS mechanisms. While this study’s limitations, including partial validation, it lays the groundwork for further investigations. Further research into RBP targets and associated regulatory pathways hold promise in unraveling the mechanisms governing testis development, spermatogenesis, and identifying potential targets for NOA treatment. This study, therefore, not only advances our fundamental understanding of human testis biology but also has significant implications for clinical practice and therapeutic development.

## Data Availability

The datasets presented in this study can be found in online repositories. The names of the repository/repositories and accession number(s) can be found in the article/**Supplementary Material**.

## Supplementary material

Supplementary Figure 1 scRNA-seq analysis of human testis from different development stages identified distinct cell types. **(A)** Dot plot showing expression of representative genes in each cell type. **(B)** Heatmap plot showing the top enrichment Gene Ontology of biological process pathways of marker genes of each cluster. **(C)** Bar plot comparing the proportions of cell populations of each cell type within each sample.

Supplementary Figure 2 Comprehensive single-cell transcriptome analysis reveals a large number of RBPs specifically expressed in different cell types of testis. **(A)** UMAP showing relative expression (z score, column scaled) levels of RBP markers of each RBP expression cell module in single-cell dataset according to cell types. Stacked bar plot showing the relative proportions of RBPs expression module in different age groups. **(B)** Stacked bar plot showing the relative proportions of RBPs expression module of each cell type in different age groups. **(C)** Bar plot showing the number of RBP in marker genes in each cell type. **(D)** Heatmap plot showing the top enrichment GO pathways of co-expressed genes of each undifferentiated SPG-specific RBP. **(E)** Stacked bar plot showing the relative proportions of RBPs expression module in different age groups.

Supplementary Figure 3 Single-cell analysis revealed heterogeneity and regulatory module of development-related RBPs in sertoli cells. **(A)** UMAP showing relative expression (z score, column scaled) levels of RBP markers of each RBP expression cell module using sertoli cells according to cell types. **(B)** Gene expression level of RBM3, NOP10, PAPK7 and FKBP1A were represented in the UMAP plot spited by different age groups.

Supplementary Figure 4 Identification of RBP modules in spermatogenesis. **(A)** UMAP showing relative expression (z score, column scaled) levels of top10 RBP markers of each cell type in germ cells. **(B)** UMAP showing relative expression (z score, column scaled) levels of top3 RBP markers of each cluster in germ cells. **(C)** UMAP showing relative expression (z score, column scaled) levels of RBP genes involved in cluster3 showed in G, according to different sperm cell types. **(D)** Cytoscape shows the co-expression networks comprising target genes selected from cluster3 associated with spermatogenesis and RBP. Edges connect RBP-target gene pairs while nodes represent genes. RBPs are displayed in red and target genes are displayed in green. Metacells of all cells were constructed and then co-expression associations of RBPs and target genes were built with Persons’ correlation analysis. Pairs with |correlation|>=0.8 and *pvalue*<=0.01 were left.

Supplementary Figure 5 Cell type-specific RBPs associated with spermatogenesis and related RAS events are significantly co-disturbed in testis tissues of patients with NOA and OA. **(A)** Bar plot showing number of detected alternative splicing events (AS) by SUVA between NOA and OA samples. **(B)** Splice junction constituting AS event detected by SUVA was annotated to classical AS event types. And the number of each classical AS event types were showed with bar plot. **(C)** Scatter plot showing the most enriched KEGG pathways of genes involved RAS with pSAR >= 50%. **(D)** Expression heatmap of all significantly differentially expressed (DE) RBPs between NOA and OA samples. **(E)** Visualization of junction reads distribution of one AS event located in STK11 in samples from NOA and OA samples. Splice junctions were labeled with SJ reads number. **(F)** The co-disturbed network among expression of overlapped RBPs showed in Figure 5 **(G)** and Figure 5 **(H)**, and splicing ratio of RAS events (pSAR>=50%) was constructed. |Pearson’s correlation| >=0.99 and pvalue <=0.01 were retained for RBP and RAS correlation. RAS involved in spermatid development and spermatogenesis terms and RBP regulators were illustrated with Cytoscape. Ellipses represent RBP. Squares in around indicate RAS.

## References

[1] Makela JA Koskenniemi JJ Virtanen HE Toppari J. Testis development. Endocr Rev. (2019) 40:857–905. doi: 10.1210/er.2018-00140 30590466

[2] O’Donnell L Smith LB Rebourcet D. Sertoli cells as key drivers of testis function. Semin Cell Dev Biol. (2022) 121:2–9. doi: 10.1016/j.semcdb.2021.06.016 34229950

[3] Rey RA. Mini-puberty and true puberty: differences in testicular function. Ann Endocrinol (Paris). (2014) 75:58–63. doi: 10.1016/j.ando.2014.03.001 24793991

[4] Chemes HE. Infancy is not a quiescent period of testicular development. Int J androl. (2001) 2001:24:2–7. doi: 10.1046/j.1365-2605.2001.00260.x 11168644

[5] Koskenniemi JJ Virtanen HE Toppari J. Testicular growth and development in puberty. Curr Opin Endocrinol Diabetes Obes. (2017) 24:215–24. doi: 10.1097/MED.0000000000000339 28248755

[6] Lucas-Herald AK Bashamboo A. Gonadal development. Endocr Dev. (2014) 27:1–16. doi: 10.1159/000363608 25247640

[7] Hernandez A Martinez ME. Thyroid hormone action in the developing testis: intergenerational epigenetics. J Endocrinol. (2020) 244:R33–46. doi: 10.1530/JOE-19-0550 PMC7220832 31977317

[8] Shah W Khan R Shah B Khan A Dil S Liu W. The molecular mechanism of sex hormones on sertoli cell development and proliferation. Front Endocrinol (Lausanne). (2021) 12:648141. doi: 10.3389/fendo.2021.648141 34367061 PMC8344352

[9] Zhou R Wu J Liu B Jiang Y Chen W Li J. The roles and mechanisms of Leydig cells and myoid cells in regulating spermatogenesis. Cell Mol Life Sci. (2019) 76:2681–95. doi: 10.1007/s00018-019-03101-9 PMC11105226 30980107

[10] Corley M Burns MC Yeo GW. How RNA-binding proteins interact with RNA: molecules and mechanisms. Mol Cell. (2020) 78:9–29. doi: 10.1016/j.molcel.2020.03.011 32243832 PMC7202378

[11] Zou D Li K Su L Liu J Lu Y Huang R. DDX20 is required for cell-cycle reentry of prospermatogonia and establishment of spermatogonial stem cell pool during testicular development in mice. Dev Cell. (2024) 59:1707–1723 e8. doi: 10.1016/j.devcel.2024.04.002 38657611

[12] Morgan M Kumar L Li Y Baptissart M. Post-transcriptional regulation in spermatogenesis: all RNA pathways lead to healthy sperm. Cell Mol Life Sci. (2021) 78:8049–71. doi: 10.1007/s00018-021-04012-4 PMC8725288 34748024

[13] Idler RK Yan W. Control of messenger RNA fate by RNA-binding proteins: an emphasis on mammalian spermatogenesis. J Androl. (2012) 33:309–37. doi: 10.2164/jandrol.111.014167 PMC5441524 21757510

[14] Sutherland JM. RNA binding proteins in spermatogenesis: an in depth focus on the Musashi family. Asian J Androl. (2015) 17:529–36. doi: 10.4103/1008-682X.151397 PMC4492041 25851660

[15] Brinegar AE Cooper TA. Roles for RNA-binding proteins in development and disease. Brain Res. (2016) 1647:1–8. doi: 10.1016/j.brainres.2016.02.050 26972534 PMC5003702

[16] Li Y Wang Y Tan Y Yue Q Guo Y Yan R. The landscape of RNA-binding proteins in mammalian spermatogenesis. Science. (2024) 386(6720):eadj8172. doi: 10.1126/science.adj8172 39208083

[17] Tang F Barbacioru C Wang Y Nordman E Lee C Xu N. mRNA-Seq whole-transcriptome analysis of a single cell. Nat Methods. (2009) 6:377–82. doi: 10.1038/nmeth.1315 19349980

[18] Guo J Grow EJ Mlcochova H Maher GJ Lindskog C Nie X. The adult human testis transcriptional cell atlas. Cell Res. (2018) 28:1141–57. doi: 10.1038/s41422-018-0099-2 PMC6274646 30315278

[19] Wang R Liu X Li L Yang M Yong J Zhai F. Dissecting human gonadal cell lineage specification and sex determination using A single-cell RNA-seq approach. Genomics Proteomics Bioinf. (2022) 20:223–45. doi: 10.1016/j.gpb.2022.04.002 PMC9684167 35513251

[20] Huang D Zuo Y Zhang C Sun G Jing Y Lei JH. A single-nucleus transcriptomic atlas of primate testicular aging reveals exhaustion of the spermatogonial stem cell reservoir and loss of Sertoli cell homeostasis. Protein Cell. (2022) 14(12):888–907. doi: 10.1093/procel/pwac057 PMC10691849 36929025

[21] Sohni A Tan K Song HW Burow D Rooij DGD Laurent L. The neonatal and adult human testis defined at the single-cell level. Cell Rep. (2019) 26:1501–1517.e4. doi: 10.1016/j.celrep.2019.01.045 30726734 PMC6402825

[22] Zhao L Yao CC Xing XY Jing T Li P Zhu Z. Single-cell analysis of developing and azoospermia human testicles reveals central role of Sertoli cells. Nat Commun. (2020) 11:5683. doi: 10.1038/s41467-020-19414-4 33173058 PMC7655944

[23] Wang M Liu XX Chang G Chen YD An G Yan LY. Single-cell RNA sequencing analysis reveals sequential cell fate transition during human spermatogenesis. Cell Stem Cell. (2018) 23:599–614 e4. doi: 10.1016/j.stem.2018.08.007 30174296

[24] Hermann BP Cheng K Singh A Cruz LRDL Mutoji KN Chen IC. The mammalian spermatogenesis single-cell transcriptome, from spermatogonial stem cells to spermatids. Cell Rep. (2018) 25:1650–1667 e8. doi: 10.1016/j.celrep.2018.10.026 30404016 PMC6384825

[25] Green CD Ma Q Manske GL Shami AN Zheng X Marini S. A comprehensive roadmap of murine spermatogenesis defined by single-cell RNA-seq. Dev Cell. (2018) 46:651–667.e10. doi: 10.1016/j.devcel.2018.07.025 30146481 PMC6713459

[26] Tang D Li K Lv M Xu C Geng H Wang C. Altered mRNAs profiles in the testis of patients with “Secondary idiopathic non-obstructive azoospermia. Front Cell Dev Biol. (2022) 10:824596. doi: 10.3389/fcell.2022.824596 35646930 PMC9133692

[27] Yang C Lin X Ji Z Huang Y Zhang L Luo J. Novel bi-allelic variants in KASH5 are associated with meiotic arrest and non-obstructive azoospermia. Mol Hum Reprod. (2022) 28(7):gaac021. doi: 10.1093/molehr/gaac021 35674372

[28] Wu X Gao S Wang L Bu T Wu S Zhou L. Role of laminin and collagen chains in human spermatogenesis - Insights from studies in rodents and scRNA-Seq transcriptome profiling. Semin Cell Dev Biol. (2022) 121:125–32. doi: 10.1016/j.semcdb.2021.07.011 34325997

[29] Chen S Wang G Zheng X Ge S Dai Y Ping P. Whole-exome sequencing of a large Chinese azoospermia and severe oligospermia cohort identifies novel infertility causative variants and genes. Hum Mol Genet. (2020) 29:2451–9. doi: 10.1093/hmg/ddaa101 32469048

[30] Voigt AL Dardari R Su L Lara NLM Sinha S Jaffer A. Metabolic transitions define spermatogonial stem cell maturation. Hum Reprod. (2022) 37:2095–112. doi: 10.1093/humrep/deac157 PMC9614685 35856882

[31] Butler A Hoffman P Smibert P Papalexi E Satija R. Integrating single-cell transcriptomic data across different conditions, technologies, and species. Nat Biotechnol. (2018) 36:411–20. doi: 10.1038/nbt.4096 PMC6700744 29608179

[32] Ianevski A Giri AK Aittokallio T. Fully-automated and ultra-fast cell-type identification using specific marker combinations from single-cell transcriptomic data. Nat Commun. (2022) 13:1246. doi: 10.1038/s41467-022-28803-w 35273156 PMC8913782

[33] Guo J Nie X Giebler M Mlcochova H Wang Y Grow EJ. The dynamic transcriptional cell atlas of testis development during human puberty. Cell Stem Cell. (2020) 26:262–276 e4. doi: 10.1016/j.stem.2019.12.005 31928944 PMC7298616

[34] Guo J Sosa E Chitiashvili T Nie X Rojas EJ Oliver E. Single-cell analysis of the developing human testis reveals somatic niche cell specification and fetal germline stem cell establishment. Cell Stem Cell. (2021) 28:764–778 e4. doi: 10.1016/j.stem.2020.12.004 33453151 PMC8026516

[35] Castello A Fischer B Eichelbaum K Horos R Beckmann BM Strein C. Insights into RNA biology from an atlas of mammalian mRNA-binding proteins. Cell. (2012) 149:1393–406. doi: 10.1016/j.cell.2012.04.031 22658674

[36] Castello A Fischer B Frese CK Horos R Alleaume AM Foehr S. Comprehensive identification of RNA-binding domains in human cells. Mol Cell. (2016) 63:696–710. doi: 10.1016/j.molcel.2016.06.029 27453046 PMC5003815

[37] Gerstberger S Hafner M Tuschl T. A census of human RNA-binding proteins. Nat Rev Genet. (2014) 15:829–45. doi: 10.1038/nrg3813 PMC11148870 25365966

[38] Hentze MW Castello A Schwarzl T Preiss T. A brave new world of RNA-binding proteins. Nat Rev Mol Cell Biol. (2018) 19:327–41. doi: 10.1038/nrm.2017.130 29339797

[39] Cao J Spielmann M Qiu X Huang X Ibrahim DM Hill AJ. The single-cell transcriptional landscape of mammalian organogenesis. Nature. (2019) 566:496–502. doi: 10.1038/s41586-019-0969-x 30787437 PMC6434952

[40] Kim D Langmead B Salzberg SL. HISAT: a fast spliced aligner with low memory requirements. Nat Methods. (2015) 12:357–60. doi: 10.1038/nmeth.3317 PMC4655817 25751142

[41] Love MI Huber W Anders S. Moderated estimation of fold change and dispersion for RNA-seq data with DESeq2. Genome Biol. (2014) 15:550. doi: 10.1186/s13059-014-0550-8 25516281 PMC4302049

[42] Cheng C Liu L Bao Y Yi J Quan W Xue Y. SUVA: splicing site usage variation analysis from RNA-seq data reveals highly conserved complex splicing biomarkers in liver cancer. RNA Biol. (2021) 18:157–71. doi: 10.1080/15476286.2021.1940037 PMC8682974 34152934

[43] Xie C Mao XZ Huang JJ Ding Y Wu JM Dong S. KOBAS 2.0: a web server for annotation and identification of enriched pathways and diseases. Nucleic Acids Res. (2011) 39:W316–22. doi: 10.1093/nar/gkr483 PMC3125809 21715386

[44] Li D Kishta MS Wang J. Regulation of pluripotency and reprogramming by RNA binding proteins. Curr Top Dev Biol. (2020) 138:113–38. doi: 10.1016/bs.ctdb.2020.01.003 PMC8015705 32220295

[45] Lin Z Tang XZ Wan J Zhang XH Liu C Liu T. Functions and mechanisms of circular RNAs in regulating stem cell differentiation. RNA Biol. (2021) 18:2136–49. doi: 10.1080/15476286.2021.1913551 PMC8632079 33896374

[46] Song H Wang L Chen D Li F. The function of pre-mRNA alternative splicing in mammal spermatogenesis. Int J Biol Sci. (2020) 16:38–48. doi: 10.7150/ijbs.34422 31892844 PMC6930371

[47] Suzuki T. Overview of single-cell RNA sequencing analysis and its application to spermatogenesis research. Reprod Med Biol. (2023) 22:e12502. doi: 10.1002/rmb2.12502 36726594 PMC9884325

[48] Chen S An G Wang HS Wu XL Ping P Hu LF. Human obstructive (postvasectomy) and nonobstructive azoospermia - Insights from scRNA-Seq and transcriptome analysis. Genes Dis. (2022) 9:766–76. doi: 10.1016/j.gendis.2020.09.004 PMC9243341 35782978

[49] Licatalosi DD. Roles of RNA-binding proteins and post-transcriptional regulation in driving male germ cell development in the mouse. Adv Exp Med Biol. (2016) 907:123–51. doi: 10.1007/978-3-319-29073-7_6 PMC6219387 27256385

[50] Wang XL Li JM Yuan SQ. Characterization of the protein expression and localization of hnRNP family members during murine spermatogenesis. Asian J Androl. (2023) 25:314–21. doi: 10.4103/aja202273 PMC10226503 36124536

[51] Qian B. RNA binding protein RBM46 regulates mitotic-to-meiotic transition in spermatogenesis. Sci Adv. (2022) 8:eabq2945. doi: 10.1126/sciadv.abq2945 36001654 PMC9401620

[52] Kleene KC. Position-dependent interactions of Y-box protein 2 (YBX2) with mRNA enable mRNA storage in round spermatids by repressing mRNA translation and blocking translation-dependent mRNA decay. Mol Reprod Dev. (2016) 83:190–207. doi: 10.1002/mrd.22616 26773323

[53] Shi B Shah W Liu L Gong CJ Zhou JT Abbas T. Biallelic mutations in RNA-binding protein ADAD2 cause spermiogenic failure and non-obstructive azoospermia in humans. Hum Reprod Open. (2023) 2023:hoad022. doi: 10.1093/hropen/hoad022 37325547 PMC10266965

[54] Fan P Muhuitijiang B Zhou J Liang H Zhang Y Zhou R. An artificial neural network model to diagnose non-obstructive azoospermia based on RNA-binding protein-related genes. Aging. (2023) 15(8):3120–40. doi: 10.18632/aging PMC10188335 37116198

[55] Kherraf ZE Cazin C Bouker A Mustapha SFB Hennebicq S Septier A. Whole-exome sequencing improves the diagnosis and care of men with non-obstructive azoospermia. Am J Hum Genet. (2022) 109:508–17. doi: 10.1016/j.ajhg.2022.01.011 PMC8948161 35172124

[56] Jiang L Li T Zhang XX Zhang BB Yu CP Li Y. RPL10L is required for male meiotic division by compensating for RPL10 during meiotic sex chromosome inactivation in mice. Curr Biol. (2017) 27:1498–1505 e6. doi: 10.1016/j.cub.2017.04.017 28502657

[57] Li H Huo YG He X Yao LP Zhang H Cui YQ. A male germ-cell-specific ribosome controls male fertility. Nature. (2022) 612:725–31. doi: 10.1038/s41586-022-05508-0 36517592

[58] Fujiwara Y Saito K Sun FY Petri S Inoue E Schimenti J. New allele of mouse DNA/RNA helicase senataxin causes meiotic arrest and infertility. Reproduction. (2023) 166:437–50. doi: 10.1530/REP-23-0166 37801077

