# Supplementary figures and images for: An integrated transcriptomic analysis unveils the regulatory roles of RNA binding proteins during human spermatogenesis
Source: Front Endocrinol (Lausanne). 2025 Feb 17;16:1522394. doi: 10.3389/fendo.2025.1522394 (PMC11872710; doi:10.3389/fendo.2025.1522394)

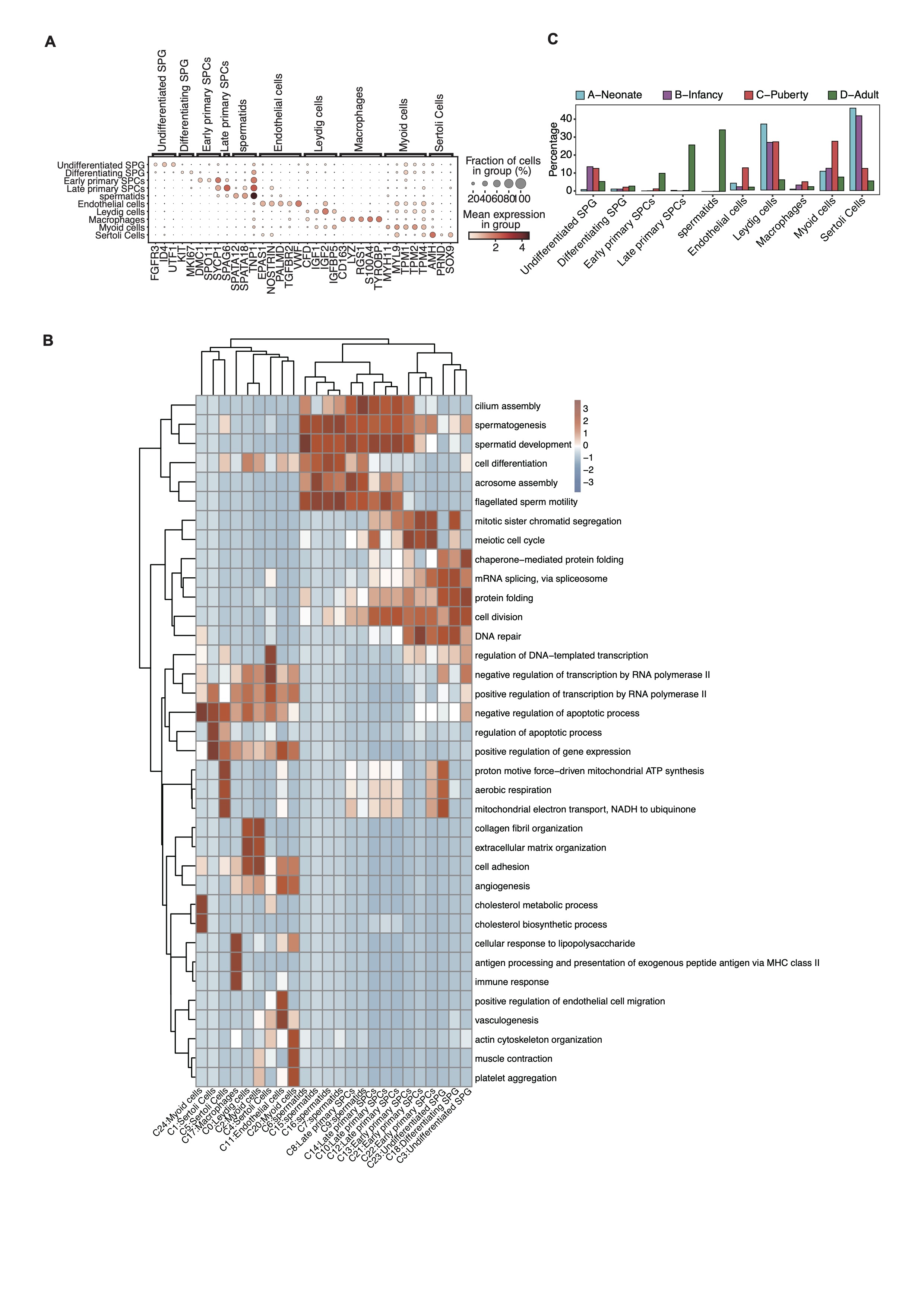

Supplement: Supplementary Figure 1 — scRNA-seq analysis of human testis from different development stages identified distinct cell types. (A) Dot plot showing expression of representative genes in each cell type. (B) Heatmap plot showing the top enrichment Gene Ontology of biological process pathways of marker genes of each cluster. (C) Bar plot comparing the proportions of cell populations of each cell type within each sample. [file Image1.jpeg]

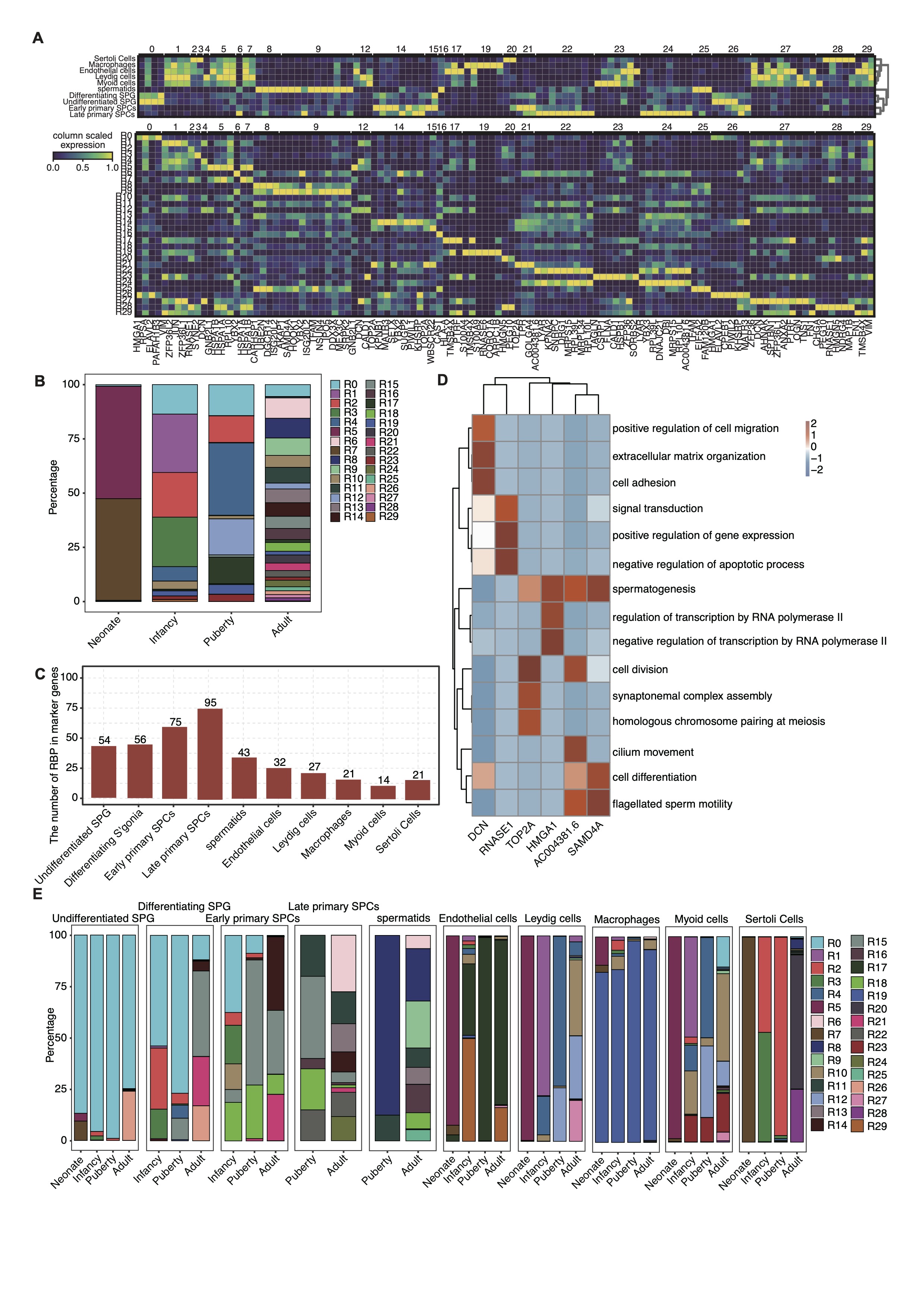

Supplement: Supplementary Figure 2 — Comprehensive single-cell transcriptome analysis reveals a large number of RBPs specifically expressed in different cell types of testis. (A) UMAP showing relative expression (z score, column scaled) levels of RBP markers of each RBP expression cell module in single-cell dataset according to cell types. Stacked bar plot showing the relative proportions of RBPs expression module in different age groups. (B) Stacked bar plot showing the relative proportions of RBPs expression module of each cell type in different age groups. (C) Bar plot showing the number of RBP in marker genes in each cell type. (D) Heatmap plot showing the top enrichment GO pathways of co-expressed genes of each undifferentiated SPG-specific RBP. (E) Stacked bar plot showing the relative proportions of RBPs expression module in different age groups. [file Image2.jpeg]

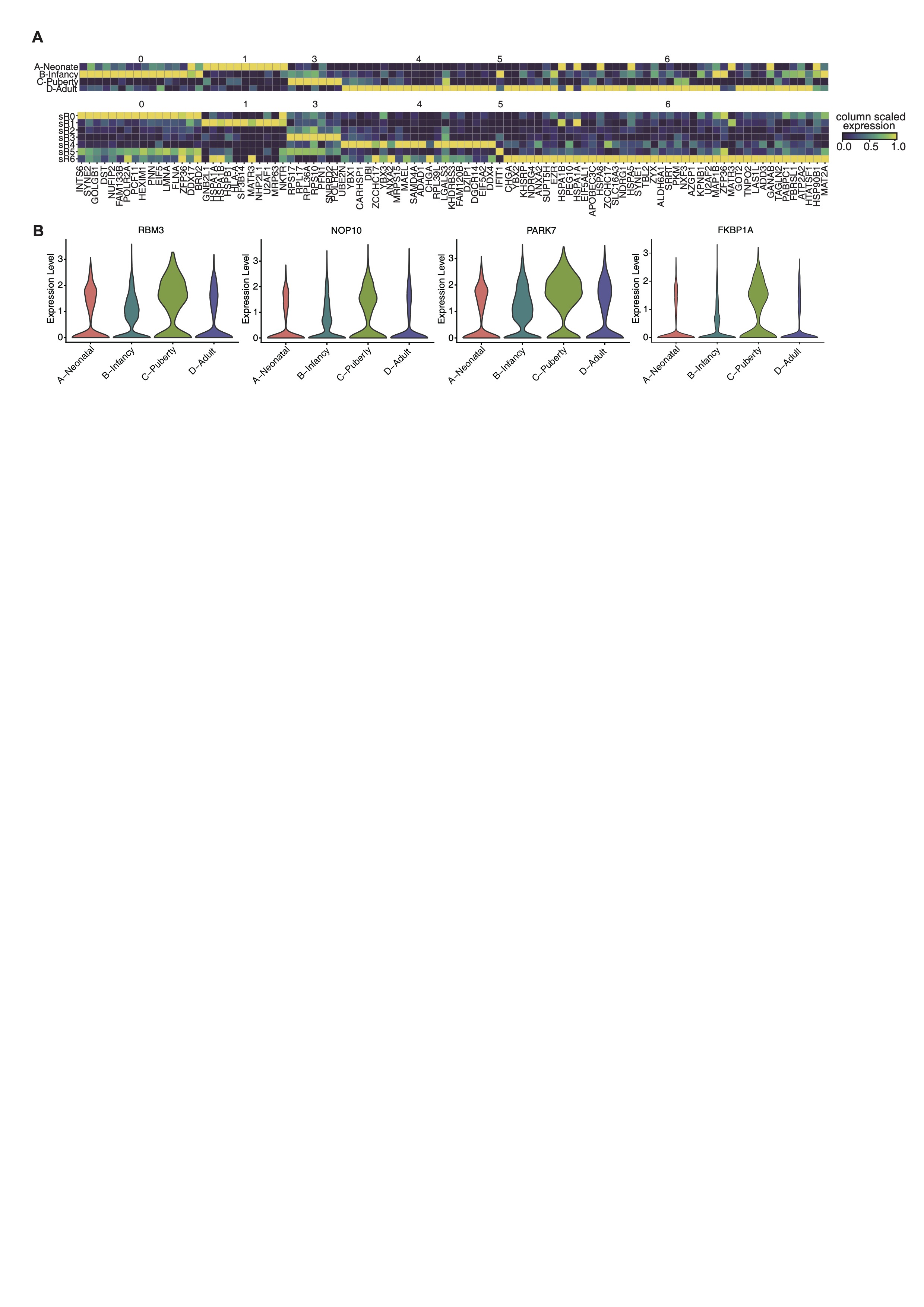

Supplement: Supplementary Figure 3 — Single-cell analysis revealed heterogeneity and regulatory module of development-related RBPs in sertoli cells. (A) UMAP showing relative expression (z score, column scaled) levels of RBP markers of each RBP expression cell module using sertoli cells according to cell types. (B) Gene expression level of RBM3, NOP10, PAPK7 and FKBP1A were represented in the UMAP plot spited by different age groups. [file Image3.jpeg]

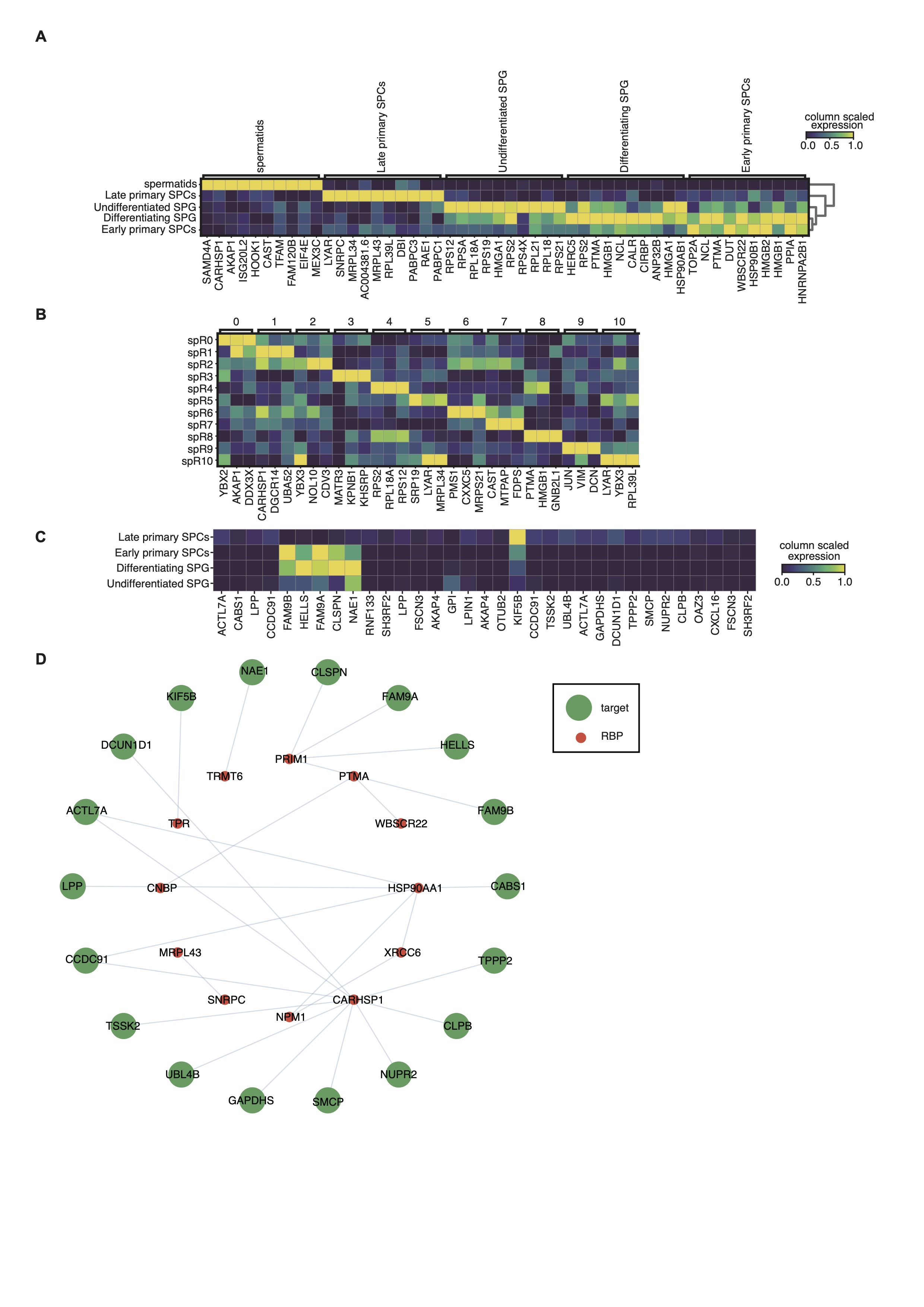

Supplement: Supplementary Figure 4 — Identification of RBP modules in spermatogenesis. (A) UMAP showing relative expression (z score, column scaled) levels of top10 RBP markers of each cell type in germ cells. (B) UMAP showing relative expression (z score, column scaled) levels of top3 RBP markers of each cluster in germ cells. (C) UMAP showing relative expression (z score, column scaled) levels of RBP genes involved in cluster3 showed in G, according to different sperm cell types. (D) Cytoscape shows the co-expression networks comprising target genes selected from cluster3 associated with spermatogenesis and RBP. Edges connect RBP-target gene pairs while nodes represent genes. RBPs are displayed in red and target genes are displayed in green. Metacells of all cells were constructed and then co-expression associations of RBPs and target genes were built with Persons’ correlation analysis. Pairs with |correlation|>=0.8 and pvalue<=0.01 were left. [file Image4.jpeg]

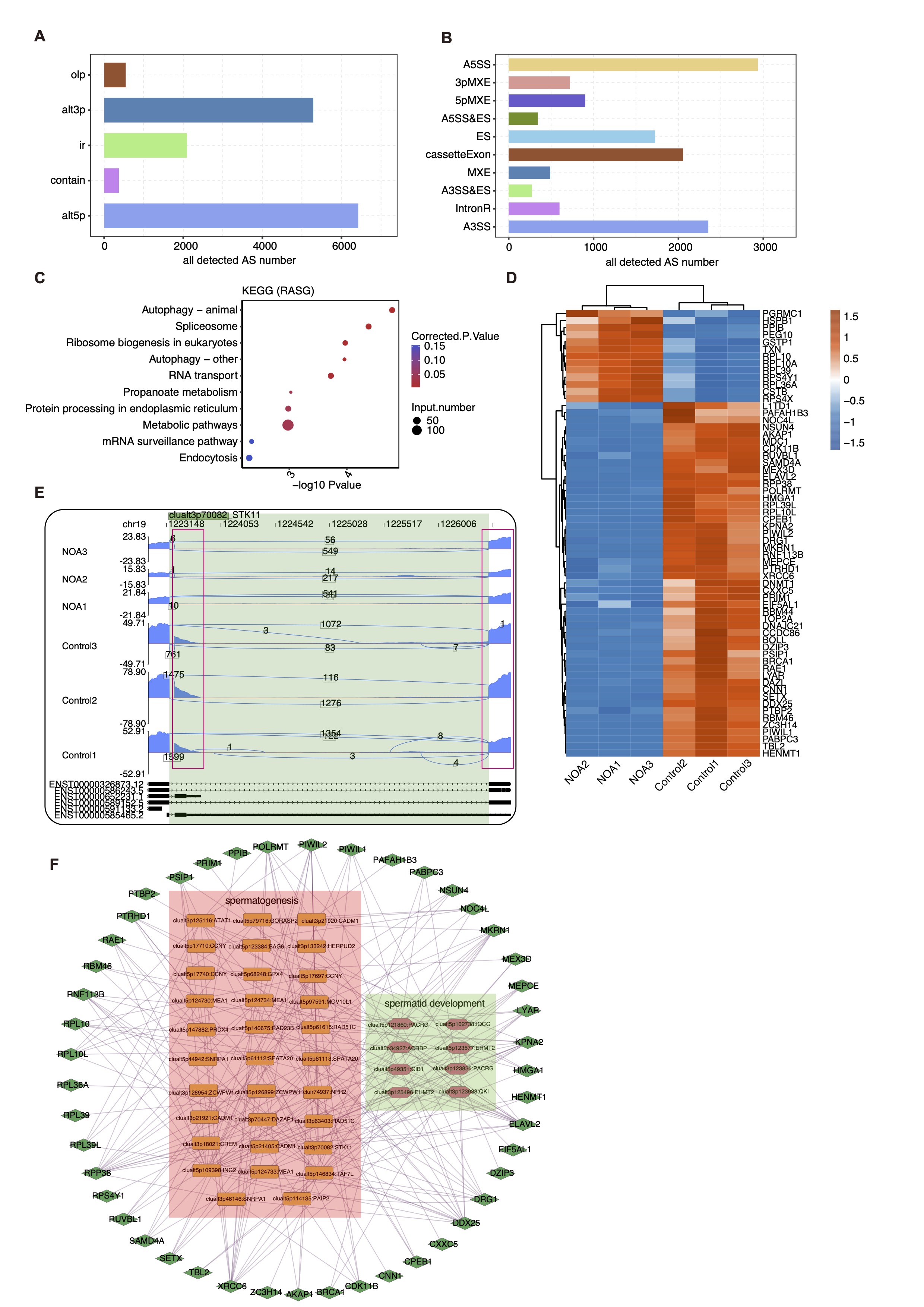

Supplement: Supplementary Figure 5 — Cell type-specific RBPs associated with spermatogenesis and related RAS events are significantly co-disturbed in testis tissues of patients with NOA and OA. (A) Bar plot showing number of detected alternative splicing events (AS) by SUVA between NOA and OA samples. (B) Splice junction constituting AS event detected by SUVA was annotated to classical AS event types. And the number of each classical AS event types were showed with bar plot. (C) Scatter plot showing the most enriched KEGG pathways of genes involved RAS with pSAR >= 50%. (D) Expression heatmap of all significantly differentially expressed (DE) RBPs between NOA and OA samples. (E) Visualization of junction reads distribution of one AS event located in STK11 in samples from NOA and OA samples. Splice junctions were labeled with SJ reads number. (F) The co-disturbed network among expression of overlapped RBPs showed in Figure 5 (G) and Figure 5 (H), and splicing ratio of RAS events (pSAR>=50%) was constructed. |Pearson’s correlation| >=0.99 and pvalue <=0.01 were retained for RBP and RAS correlation. RAS involved in spermatid development and spermatogenesis terms and RBP regulators were illustrated with Cytoscape. Ellipses represent RBP. Squares in around indicate RAS. [file Image5.jpeg]
